# Development and internal validation of an early inpatient risk score for in-hospital mortality in acute pancreatitis: A retrospective cohort study

**DOI:** 10.1371/journal.pone.0352980

**Published:** 2026-07-06

**Authors:** Abdulrahman Al-Dawoudi, Daniil Varlamov, Mujahed Dalain, Nazar Kopytko, Aiga Staka, Davis Freimanis

**Affiliations:** 1 Faculty of Medicine and Life Sciences, University of Latvia, Riga, Latvia; 2 Medical Faculty 1, Poltava State Medical University, Poltava, Ukraine; 3 Department of Gastroenterology, Pauls Stradins Clinical University Hospital, Riga, Latvia; Charite Universitatsmedizin Berlin, GERMANY

## Abstract

**Introduction:**

Early identification of patients at high risk of mortality remains a clinical challenge in acute pancreatitis. Existing prognostic tools are often complex or require repeated assessments, limiting their routine use. We aimed to develop and internally validate an early admission-phase risk score for predicting in-hospital mortality in patients with acute pancreatitis.

**Methods:**

We conducted a retrospective cohort study of consecutive adult patients admitted with acute pancreatitis between 2020 and 2024. Acute pancreatitis was defined according to the revised Atlanta criteria. Multivariable logistic regression was used to develop a prediction model for in-hospital mortality using routinely available demographic, clinical, and laboratory variables obtained within the first 24 hours of hospitalization. Missing data were handled using multiple imputation. Model performance was assessed by discrimination and calibration, with internal validation performed using bootstrap resampling and temporal validation conducted in patients admitted during 2023–2024.

**Results:**

A total of 1,041 patients were included, of whom 53 (5.1%) died during hospitalization. The final model incorporated age, early multi-organ dysfunction within 24 hours of admission, C-reactive protein, and urea levels. The model showed good discrimination (area under the receiver operating characteristic curve, 0.81) and good agreement between predicted and observed in-hospital mortality probabilities. Bootstrap internal validation showed minimal optimism, and temporal validation in patients admitted during 2023–2024 confirmed stable model performance. A simplified risk score derived from the model stratified patients into low-to-intermediate-risk and high-risk categories, with substantially higher observed in-hospital mortality in the high-risk group.

**Conclusions:**

We developed and internally validated an admission-phase risk score for predicting in-hospital mortality in patients with acute pancreatitis using readily available clinical variables. The proposed score may support early inpatient risk stratification within the first 24 hours of hospitalization. However, external validation in independent cohorts across diverse healthcare settings, patient populations, and pancreatitis etiologies is required before broader clinical application.

## Introduction

The global incidence of acute pancreatitis has been steadily increasing, with significant variability across European countries, ranging from 4.6 to 100 per 100,000 population. Multiple studies across Europe have reported an upward trend in incidence, with a median annual increase of 3.4% [1–4]. This trend highlights the growing burden of acute pancreatitis, driven by factors like metabolic diseases, alcohol consumption, and other regional risk factors. Approximately 20% to 30% of patients progress to moderately severe or severe forms of the disease, with mortality rates reaching up to 30% in cases of infected pancreatic necrosis [5–7].

Several prognostic tools have been developed to assess risk in acute pancreatitis. The BISAP score, a five-variable tool assessable within 24 hours, offers high specificity (~90%) but moderate sensitivity (~50–60%) for mortality prediction [8]. Ranson’s criteria, while historically useful, require 48-hour data, which delays risk stratification. Meta-analytic evidence suggests that while Ranson’s criteria offer greater sensitivity, they lack the specificity of BISAP [9]. A major limitation of many existing systems, including APACHE II, is their complexity and reliance on numerous variables, which limits their practical applicability in the modern management of acute pancreatitis [10,11]. Moreover, recent machine learning models, although promising, face challenges in design, validation, and reporting, further underscoring the need for simpler, more clinically feasible stratification tools [12].

In response, we developed a simplified early inpatient risk score based on a limited number of routinely available clinical and laboratory variables assessed within the first 24 hours of hospitalization. By avoiding complex or resource-intensive assessments, the proposed score aims to facilitate practical identification of patients at increased risk of in-hospital mortality during the initial hospitalization period. The objective of this study was to develop and internally validate a points-based risk score for predicting in-hospital mortality in adults with acute pancreatitis, with prospective evaluation required to determine its impact on clinical outcomes or healthcare processes.

## Materials and methods

### Study design and setting

This was a single-center, retrospective cohort study conducted at Pauls Stradiņš Clinical University Hospital in Riga, Latvia. The objective of this study was to develop and internally validate an early admission-phase clinical risk score for predicting in-hospital mortality using routinely available demographic, clinical, and laboratory data obtained within the first 24 hours of hospitalization. In this study, the term ‘admission-phase’ refers to variables routinely available during the initial hospitalization period, defined as within the first 24 hours of hospital admission.

### Study population and eligibility criteria

All consecutive adult patients (≥18 years) admitted with a primary diagnosis of acute pancreatitis between January 1, 2020, and December 31, 2024, were screened for eligibility. Acute pancreatitis was diagnosed according to the revised Atlanta criteria, requiring at least two of the following: (1) abdominal pain consistent with acute pancreatitis; (2) serum lipase or amylase levels ≥3 times the upper limit of normal; and (3) imaging findings consistent with acute pancreatitis on contrast-enhanced computed tomography or transabdominal ultrasound [13,14].

#### Exclusion criteria.

**Fig 1:**
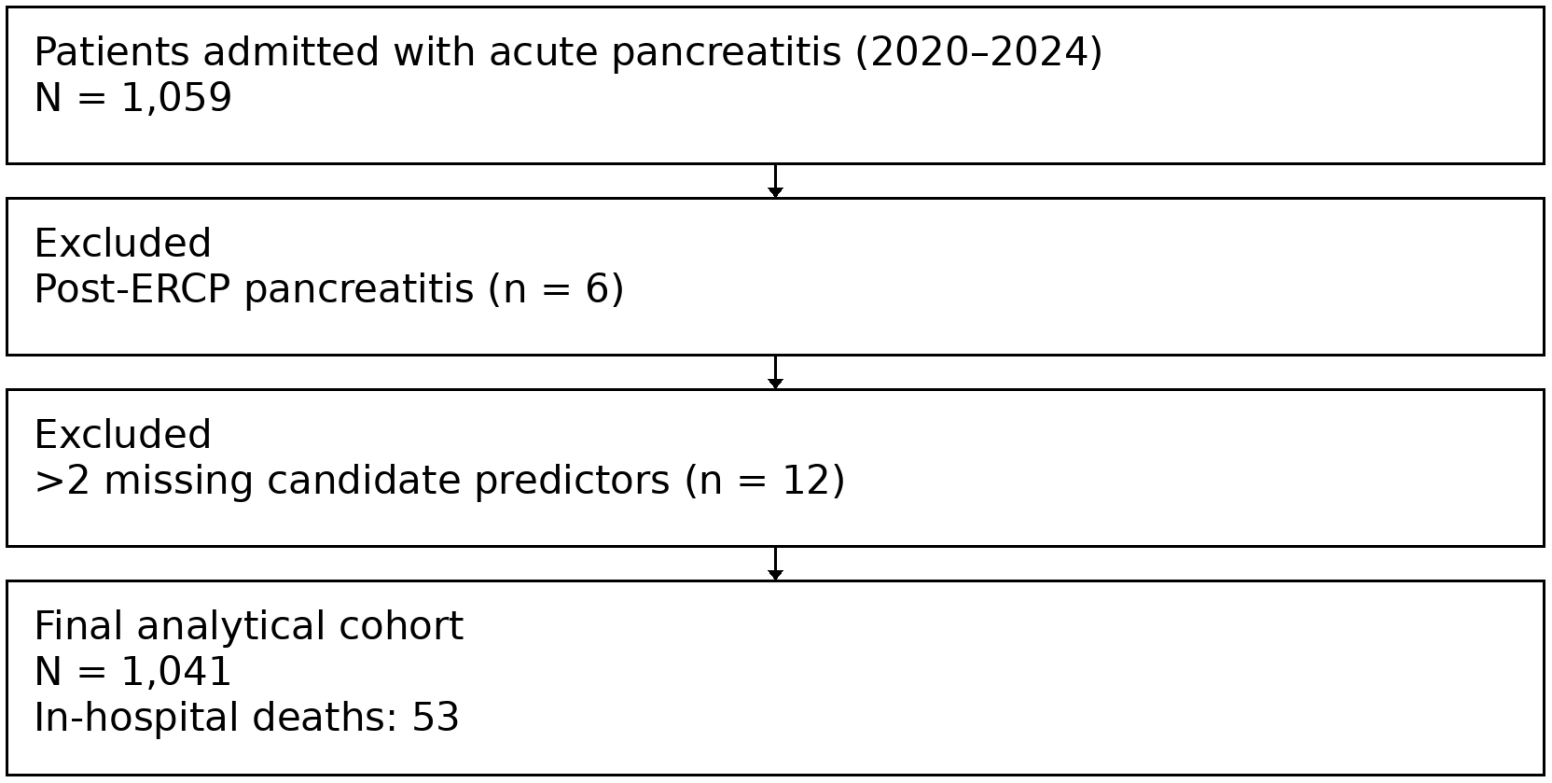
Study flow diagram. Flow diagram illustrating patient selection and exclusions for the acute pancreatitis cohort admitted between 2020 and 2024. Of 1,059 initially identified patients, 6 with post–endoscopic retrograde cholangiopancreatography pancreatitis and 12 with missing data for more than two candidate predictor variables were excluded. The final analytical cohort comprised 1,041 patients, including 53 in-hospital deaths.

Patients with chronic pancreatitis were excluded during data extraction. Patients were further excluded if they had post–endoscopic retrograde cholangiopancreatography (post-ERCP) pancreatitis or missing data for the primary outcome (in-hospital mortality). Patients with missing data for more than two candidate predictor variables were excluded due to insufficient data completeness. Patients with missing values in two or fewer candidate predictors were retained and included in the analysis using multiple imputation. The patient selection process and exclusions are summarized in Fig 1.

#### Outcomes.

The primary outcome was in-hospital mortality, defined as death from any cause occurring during the index hospitalization for acute pancreatitis.

### Predictor variables

All predictor variables were defined using data available within the first 24 hours of hospitalization.

- Age (years): Age was modeled as a continuous variable in regression analyses and operationalized for the risk score as decade units by dividing age in years by 10 and rounding down to the nearest whole number, with 1 point assigned per decade. This approach improves clinical interpretability while preserving prognostic information.
- Sex (male/female).

#### Clinical variable.

Early multi-organ dysfunction syndrome (MODS): Defined using the modified Marshall scoring system and assessed within the first 24 hours of hospital admission. MODS was considered present if the Marshall score was ≥ 2 in at least one organ system [15]. Although organ dysfunction may evolve during this early period, assessment within the first 24 hours reflects routine early inpatient evaluation and captures clinically relevant physiological deterioration occurring at or shortly after presentation. Accordingly, early MODS was treated as an early inpatient predictor reflecting physiological deterioration identified during the first 24 hours of hospitalization. The model is intended for risk stratification during this early inpatient period and not for immediate point-of-arrival or emergency department triage prediction.

#### Laboratory variables.

The first measured laboratory values after hospital admission were collected:

- C-reactive protein (CRP, mg/L)
- Creatinine (μmol/L)
- White blood cell count (WBC, × 10⁹/L)
- Alanine aminotransferase (ALT, U/L)
- Glucose (mmol/L)
- Sodium (mmol/L)
- Urea (mmol/L)
- Hematocrit (Hct, %)

Admission to the intensive care unit (ICU) was not included as a predictor variable, as it represents a post-admission management decision rather than a baseline patient characteristic.

### Data collection and management

Patient data were extracted from the hospital’s electronic medical records and anonymized using unique study identification codes. Baseline characteristics were summarized using observed, non-imputed values. Missing predictor data were present in 54 of 1,041 patients (5.2%). Missing values occurred for urea in 45 patients (4.3%), glucose in 10 patients (1.0%), and alanine aminotransferase in 2 patients (0.2%); all remaining candidate predictors were complete. No values were missing for the primary outcome of in-hospital mortality. Missing predictor values were handled using multiple imputation with chained equations, generating 20 imputed datasets. The imputation model included all candidate predictors, in-hospital mortality, and admission year. Because the outcome variable was complete, it was included as a predictor in the imputation model but was not itself imputed. The imputed datasets were used for regression analyses and model performance assessment.

### Sample size considerations

A total of 1,041 patients met the inclusion criteria, yielding 53 in-hospital mortality events (5.1%). Eleven candidate predictor parameters were evaluated during model development, corresponding to 4.82 events per candidate predictor parameter. Given this limited event burden and the associated potential for overfitting and imprecision in coefficient estimates [16], model robustness was assessed using bootstrap internal validation [17] and an additional ridge-penalized regression sensitivity analysis incorporating all candidate predictors.

### Ethical considerations

The study was approved by the Medical Research Ethics Committee of the Faculty of Medicine and Life Sciences, University of Latvia (approval No 23-37/301). All procedures were conducted in accordance with the Declaration of Helsinki. Patient data were fully anonymized prior to analysis, and informed consent was waived in accordance with local regulations due to the retrospective cohort design. The dataset was accessed for research purposes on 10 December 2025.

### Statistical analysis

All statistical analyses were performed using SPSS Statistics version 28.0 (IBM Corp., Armonk, NY, USA) and R software (version 4.5.x; R Foundation for Statistical Computing, Vienna, Austria). Descriptive statistics and univariable and multivariable logistic regression analyses were performed using SPSS, while model performance evaluation and internal validation were conducted in R using RStudio (version 2026.01.0 + 392; Posit Software, PBC).

Given the limited number of outcome events, split-sample validation was not performed. Instead, internal validation was conducted using bootstrap resampling to assess model optimism.

Temporal validation was performed using data from later admission years within the same single-center cohort.

#### Phase 1: Data preparation.

- Missing data: Multiple imputation was applied for predictor variables with <10% missing values.
- Linearity: The linearity assumption for continuous predictors was evaluated using restricted cubic splines.

#### Phase 2: Model development.

- Univariable analysis: The initial candidate predictor set comprised 11 variables: age, sex, early multi-organ dysfunction within 24 hours, C-reactive protein, creatinine, white blood cell count, alanine aminotransferase, glucose, sodium, urea, and hematocrit. These variables were selected for evaluation because they are routinely available within the first 24 hours of hospitalization and represent clinically relevant domains, including demographic characteristics, early organ dysfunction, systemic inflammation, renal and metabolic abnormalities, and hemoconcentration, which have previously been associated with severity or mortality in acute pancreatitis [18–24]. Univariable logistic regression was used to assess associations between candidate predictors and in-hospital mortality. Predictors demonstrating statistical significance (p < 0.05) and clinical relevance were considered for multivariable modeling. Odds ratios (ORs) with 95% confidence intervals (CIs) were reported.
- Multivariable analysis: A multivariable logistic regression model was constructed using candidate predictors identified in univariable analyses. Backward stepwise selection guided by the Akaike Information Criterion (AIC) was used to inform predictor retention. Following this selection-stage analysis, creatinine was excluded because it was not independently associated with mortality, and the final parsimonious continuous prediction model retained age, early multi-organ dysfunction within 24 hours, C-reactive protein, and urea. Model complexity was constrained to limit overfitting, supported by internal validation.
- Penalized-regression sensitivity analysis. To address potential overfitting associated with univariable screening followed by backward stepwise selection, we performed an additional sensitivity analysis using ridge-penalized logistic regression incorporating all 11 candidate predictors: age, sex, early multi-organ dysfunction within 24 hours, C-reactive protein, creatinine, white blood cell count, alanine aminotransferase, glucose, sodium, urea, and hematocrit. The analysis was conducted in the full analytical cohort using the same multiple-imputation framework as the primary analysis. The ridge penalty parameter was selected by 10-fold cross-validation in the multiply imputed analytical cohort. Performance of the ridge-penalized model was compared with that of the existing four-predictor continuous model underlying the simplified score using optimism-corrected area under the receiver operating characteristic curve and Brier score obtained through 1,000 bootstrap resamples.
- Multicollinearity: Variance inflation factors (VIFs) were calculated, with VIF < 5 considered acceptable.

### Risk score development

Following development of the multivariable prediction model, a simplified points-based clinical score was constructed to facilitate bedside use. Age, C-reactive protein, and urea were retained as continuous variables in the underlying regression model used for prediction. For construction of the simplified score, age was represented in completed 10-year increments, while C-reactive protein and urea were categorized using thresholds of ≥100 mg/L and ≥8 mmol/L, respectively. These thresholds were selected pragmatically to represent clinically relevant inflammatory and renal and metabolic abnormalities during the early inpatient period and were not derived through statistical cut-point optimization. The simplified score assigned 1 point per completed decade of age, 4 points for early multi-organ dysfunction within 24 hours, 2 points for C-reactive protein ≥100 mg/L, and 2 points for urea ≥8 mmol/L. This points-based tool was developed as a pragmatic clinical simplification of the underlying continuous model to support bedside risk stratification and should not be interpreted as an exact representation of model-derived predicted probabilities.

Individual patient risk scores were calculated by summing all assigned component points. The complete early inpatient risk score tool, including scoring instructions and risk-group classification, is provided in the Supporting Information (S2 File). For descriptive risk grouping, patients with a total score ≥6 points were classified as high risk, whereas those with a total score ≤5 points were classified as low-to-intermediate risk.

### Model validation and performance assessment

#### Internal validation.

We performed internal validation using bootstrap resampling with 1,000 iterations to assess model stability and obtain optimism-corrected estimates of model performance.

#### Discrimination.

Model discrimination was evaluated using the area under the receiver operating characteristic curve (AUC).

#### Calibration.

Calibration was assessed using calibration plots, as well as calculation of the calibration slope and intercept.

#### Overall performance.

Overall predictive accuracy was quantified using the Brier score.

#### Sensitivity analyses.

- Complete-case analysis: A complete-case analysis was conducted to assess the impact of missing data handling on model performance.
- MODS-exclusion sensitivity analysis: A secondary sensitivity analysis excluding early multi-organ dysfunction within the first 24 hours as a predictor from the model was performed to evaluate whether model performance was dependent on inclusion of this evolving disease-severity variable.
- Temporal validation: Temporal validation was performed using data from later admission years (2023–2024) to evaluate model performance in a more recent patient cohort.

Decision curve analysis was performed to evaluate the clinical usefulness of the prediction model by quantifying net benefit across a range of threshold probabilities.

Reporting of this study was guided by the TRIPOD (Transparent Reporting of a multivariable prediction model for Individual Prognosis Or Diagnosis) statement (S1 Checklist).

## Results

Among the 1,059 patients initially identified, 6 were excluded due to post–endoscopic retrograde cholangiopancreatography pancreatitis, and 12 were excluded because of missing data for more than two predictor variables. The final analytical cohort included 1,041 patients, with 53 in-hospital deaths.

Baseline characteristics of the study population stratified by in-hospital mortality are shown in Table 1. Compared with survivors, patients who died during hospitalization were older and had higher levels of inflammatory and renal biomarkers during the first 24 hours of hospitalization, particularly C-reactive protein, creatinine, and urea. Early multi-organ dysfunction within the first 24 hours was also more frequent among deceased patients. Unadjusted between-group comparisons are reported in Table 1.

**Table 1:** Baseline characteristics of the study population according to in-hospital mortality.

Variable	Alive (n = 988)	Deceased (n = 53)	Total (n = 1,041)	p-value
**Age, years**	50.0 (39.0–64.0)	60.0 (51.0–67.0)	50.0 (39.0–64.0)	0.002
**CRP, mg/L**	15.3 (4.0–68.8)	78.2 (8.0–266.9)	16.2 (4.0–74.7)	<0.001
**Creatinine, µmol/L**	76.0 (63.0–94.0)	130.0 (93.0–236.0)	77.0 (64.0–96.0)	<0.001
**WBC, × 10⁹/L**	11.1 (8.3–14.1)	14.5 (9.7–18.2)	11.2 (8.3–14.3)	<0.001
**ALT, U/L**	62.0 (29.0–160.8)	77.0 (44.0–135.0)	63.0 (29.0–160.0)	0.241
**Glucose, mmol/L**	6.8 (5.8–8.7)	8.2 (6.5–10.7)	6.9 (5.8–8.8)	0.002
**Sodium, mmol/L**	137.0 (134.0–140.0)	135.0 (131.0–139.0)	137.0 (134.0–140.0)	0.036
**Urea, mmol/L**	4.8 (3.4–6.9)	10.4 (6.2–16.2)	5.0 (3.5–7.2)	<0.001
**Hematocrit, %**	41.6 (37.2–44.8)	43.0 (37.2–46.2)	41.6 (37.2–44.8)	0.082
**Male sex**	616 (62.3%)	33 (62.3%)	649 (62.3%)	1.000
**MODS <24 h**	3 (0.3%)	9 (17.0%)	12 (1.2%)	<0.001

Continuous variables are presented as median (interquartile range) and were compared between survivors and deceased patients using the Mann–Whitney U test. Categorical variables are presented as number (%) and were compared using Fisher’s exact test. Comparisons were based on observed, non-imputed values; missing values were excluded on a variable-specific basis.

In univariable logistic regression analyses, increasing age, early multi-organ dysfunction within 24 hours, and early inpatient levels of inflammatory and metabolic biomarkers, including C-reactive protein, creatinine, white blood cell count, glucose, and urea, were associated with increased odds of in-hospital mortality (Table 2). Sex, alanine aminotransferase, sodium, and hematocrit were not significantly associated with in-hospital mortality in univariable analyses.

**Table 2:** Univariable logistic regression analysis for in-hospital mortality.

Predictor	Odds ratio (OR)	95% CI	p-value
**Age (per year)**	1.023	1.007–1.045	0.006
**Male sex**	1.00	0.56–1.76	0.99
**MODS <24 h**	67.16	22.60–199.60	<0.001
**CRP (per mg/L)**	1.006	1.004–1.009	<0.001
**Creatinine (per µmol/L)**	1.005	1.003–1.007	<0.001
**WBC (per ×10⁹/L)**	1.103	1.056–1.153	<0.001
**ALT (per U/L)**	1.000	0.999–1.001	0.430
**Glucose (per mmol/L)**	1.074	1.027–1.123	0.002
**Sodium (per mmol/L)**	0.959	0.909–1.012	0.120
**Urea (per mmol/L)**	1.154	1.108–1.202	<0.001
**Hematocrit (per %)**	1.012	0.968–1.058	0.600

Odds ratios (ORs) are reported with 95% confidence intervals (CIs). Continuous variables are modeled per unit increase as specified.

In the multivariable logistic regression model, increasing age, early multi-organ dysfunction within the first 24 hours of admission, and higher early inpatient levels of C-reactive protein and urea were independently associated with increased odds of in-hospital mortality (Table 3). Creatinine was not independently associated with mortality after adjustment for other variables. Accordingly, creatinine was excluded from the final four-predictor continuous prediction model and from the derived simplified points-based score; Table 3 is presented to document the multivariable predictor-selection process. Although white blood cell count and glucose were associated with mortality in univariable analyses, they were not retained in the AIC-guided multivariable selection procedure.

**Table 3:** Multivariable logistic regression analysis informing predictor selection for the simplified risk score.

Predictor	Adjusted OR	95% CI	p-value
**Age (per year)**	1.023	1.001–1.045	0.047
**MODS <24 h**	22.61	4.87–104.89	<0.001
**CRP (per mg/L)**	1.006	1.003–1.009	<0.001
**Creatinine (per μmol/L)**	1.000	0.998–1.002	0.452
**Urea (per mmol/L)**	1.08	1.01–1.14	0.018

Odds ratios (ORs) are reported with 95% confidence intervals (CIs). Continuous variables are modeled per unit increase as specified. For variables with small per-unit effect sizes (e.g., CRP), estimates are presented with three-decimal precision to avoid rounding artifacts.

### Risk classification performance

For the underlying continuous prediction model, classification performance was additionally evaluated at a predicted-probability threshold of 0.05. At this threshold, the model achieved a sensitivity of 67.9% and a specificity of 79.55%. The 0.05 threshold was selected for descriptive performance reporting based on the observed baseline in-hospital mortality rate and clinical considerations, rather than through formal statistical optimization; it was not intended to define a clinical decision-making cut-off or the points-based risk-group threshold described below.

Following development of the continuous multivariable prediction model, a simplified points-based clinical score was constructed for bedside use, as described in the Methods (Table 4). Creatinine was not incorporated into the simplified score because it was not independently associated with mortality after multivariable adjustment. C-reactive protein and urea remained continuous in the underlying regression model and were categorized only for construction of the simplified bedside score.

**Table 4:** Risk score construction for prediction of in-hospital mortality.

Predictor	Criteria	Points
**Age**	Per 10-year increase	1
**MODS <24 h**	Present	4
**CRP**	≥100 mg/L	2
**Urea**	≥8 mmol/L	2

The observed total risk score range in the study population was 1–15 points. The theoretical maximum score is not fixed, as age contributes incrementally without an upper cap within the model structure.

### Model discrimination

**Fig 2:**
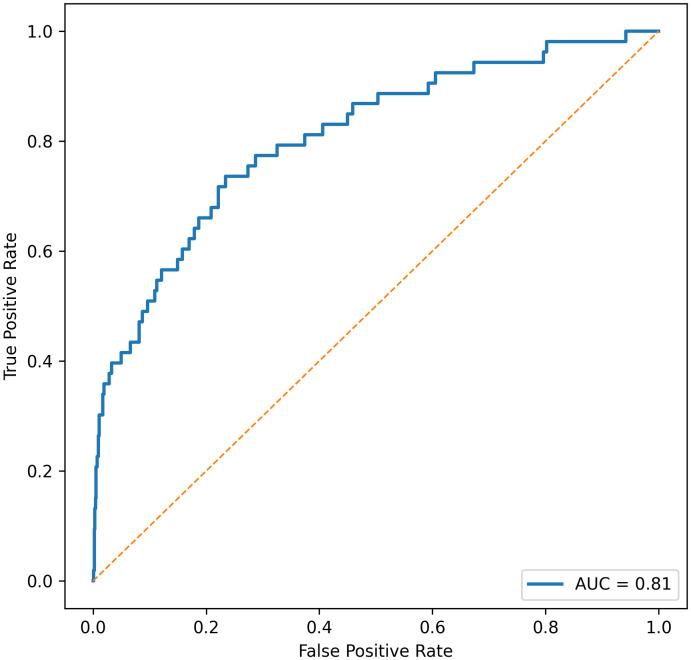
Receiver operating characteristic (ROC) curve of the final prediction model for in-hospital mortality.

The discriminative performance of the final prediction model was evaluated using receiver operating characteristic (ROC) curve analysis. The model achieved good discrimination for in-hospital mortality, with an area under the ROC curve (AUC) of 0.81 (95% CI 0.74–0.88) (Fig 2).

Illustrating good discriminative performance in the full study cohort (AUC = 0.81, 95% CI 0.74–0.88). The dashed diagonal line represents the line of no discrimination.

### Model calibration

**Fig 3:**
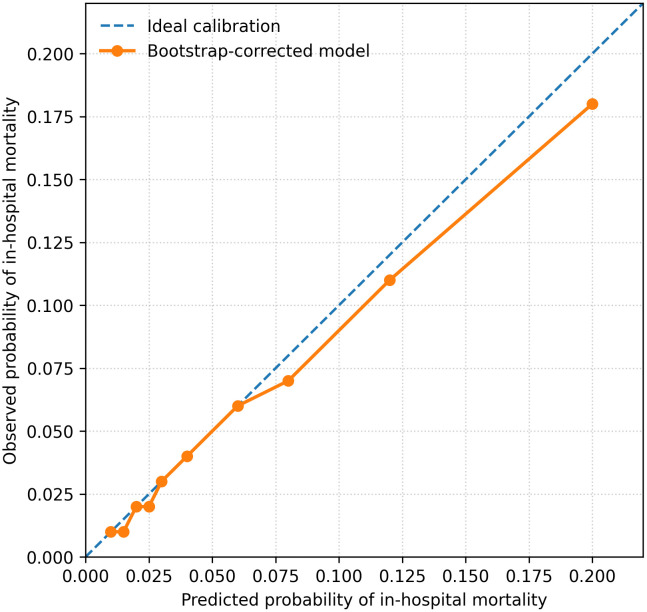
Calibration plot of the final prediction model for in-hospital mortality. Predicted and observed in-hospital mortality probabilities across deciles of predicted risk are shown. The solid line represents the bootstrap-corrected calibration curve, and the dashed line indicates ideal calibration. Most observations fall within lower predicted risk ranges, reflecting the low overall event rate.

Model calibration was assessed using a calibration plot comparing predicted and observed in-hospital mortality probabilities (Fig 3). The model demonstrated good agreement between predicted and observed risks across the range of clinically relevant predicted probabilities.

The bootstrap optimism-corrected calibration intercept was 0.00 (95% CI −0.73 to 0.73), indicating no evidence of systematic over- or underestimation of risk, and the bootstrap optimism-corrected calibration slope was 1.00 (95% CI 0.75 to 1.25), suggesting adequate agreement between predicted and observed probabilities without clear evidence of substantial overfitting. The optimism-corrected Brier score was 0.0410, indicating good overall predictive accuracy.

### Decision curve analysis

**Fig 4:**
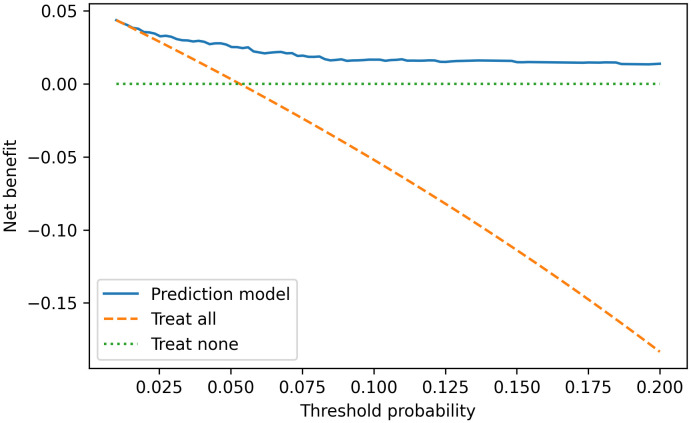
Decision curve analysis of the prediction model for in-hospital mortality. Net benefit of the prediction model compared with treat-all and treat-none strategies across a range of clinically relevant threshold probabilities, demonstrating potential utility for early risk stratification.

Decision curve analysis demonstrated that the model provided a higher net benefit than treat-all and treat-none strategies across clinically relevant threshold probabilities ranging from approximately 2% to at least 15%, supporting potential utility for early inpatient risk stratification (Fig 4).

Applying the descriptive risk-group classification rule, in-hospital mortality was substantially higher in the high-risk group than in the low-to-intermediate-risk group (8.8% vs 1.8%, respectively; Table 5). The ≥ 6-point threshold was selected based on inspection of the score distribution and corresponding observed mortality across score levels; formal sensitivity–specificity optimization was not performed to avoid overfitting given the limited number of outcome events.

**Table 5:** Risk stratification according to the derived mortality risk score.

Risk group	Score range	Patients (n)	Deaths (n)	In-hospital mortality (%)
**Low-to-intermediate risk**	≤5	550	10	1.8%
**High risk**	≥6	491	43	8.8%
**Total**	—	1,041	53	5.1%

Low- and intermediate-risk categories were combined due to the low number of outcome events in the lowest-risk group. High risk was defined as a total score ≥6 points. In-hospital mortality is reported as the proportion of deaths within each risk group.

### Internal validation

Internal validation was performed using bootstrap resampling with 1,000 iterations to assess model optimism. The optimism-corrected area under the receiver operating characteristic curve was 0.80, indicating minimal overfitting and stable model performance.

### Internal temporal validation

Internal temporal validation was performed in patients admitted during 2023–2024. In this subset, the model maintained preserved discriminative performance, with an area under the receiver operating characteristic curve of 0.83 and a Brier score of 0.037. Calibration remained acceptable, with a bootstrap optimism-corrected calibration intercept of 0.00 (95% CI −1.31 to 1.31) and a bootstrap optimism-corrected calibration slope of 1.00 (95% CI 0.56 to 1.44), indicating stable agreement between predicted and observed risks in the more recent cohort.

### Sensitivity analyses

Sensitivity analyses were performed to assess the robustness of the prediction model. A complete-case analysis excluding patients with missing predictor data yielded similar discriminative performance compared with the primary analysis. To address potential concerns regarding incorporation of early disease severity, an additional sensitivity analysis was conducted excluding early multi-organ dysfunction (MODS <24 h) from the model. In this analysis, the model retained acceptable discriminative performance, with an area under the receiver operating characteristic curve of 0.79 and a Brier score of 0.044. Calibration remained satisfactory, with a bootstrap optimism-corrected calibration intercept of 0.00 (95% CI −0.71 to 0.71) and a bootstrap optimism-corrected calibration slope of 1.00 (95% CI 0.75 to 1.25), indicating that predictive performance was not solely driven by inclusion of early organ dysfunction.

Penalized-regression sensitivity analysis. An additional modeling-strategy sensitivity analysis was conducted in the full analytical cohort of 1,041 patients, including all 53 in-hospital deaths, using 20 imputed datasets. The existing four-predictor continuous model underlying the simplified score achieved an apparent AUC of 0.808 and an optimism-corrected AUC of 0.799, with an optimism-corrected Brier score of 0.0410. The ridge-penalized model incorporating all 11 candidate predictors achieved an apparent AUC of 0.841 and an optimism-corrected AUC of 0.814, with an optimism-corrected Brier score of 0.0418. Thus, ridge penalization produced a modest increase in corrected discrimination of 0.015 but did not improve overall predictive accuracy as assessed by the Brier score. Given its greater parsimony and clinical interpretability, the original simplified scoring tool was retained.

## Discussion

In this retrospective cohort analysis, we developed and internally validated an admission-phase clinical risk score for predicting in-hospital mortality among adults hospitalized with acute pancreatitis. Using routinely available variables obtained within the first 24 hours of hospitalization, the model demonstrated good discrimination, good calibration, and stable performance across multiple internal validation strategies, including bootstrap resampling, complete-case analysis, and temporal validation in a more recent patient cohort. Collectively, these findings suggest that the proposed score may support early inpatient risk stratification within the first 24 hours of hospitalization. Potential clinical applications include supporting early inpatient risk stratification and identifying patients who may warrant closer clinical observation during the initial hospitalization period.

For the underlying continuous prediction model, a predicted-probability threshold of 0.05 was used for descriptive classification-performance reporting, yielding a sensitivity of 67.9% and a specificity of 79.55%; this threshold was separate from the ≥ 6-point threshold used for the simplified bedside score. This threshold reflects a pragmatic balance between sensitivity and specificity for early inpatient risk stratification and was informed by the observed baseline in-hospital mortality rate rather than formal statistical optimization. While alternative thresholds could increase sensitivity at the expense of specificity, the 0.05 threshold was chosen to illustrate model performance and risk stratification characteristics without implying a prescriptive clinical action or decision-making cut-off.

The final model incorporated age, early multi‑organ dysfunction within the first 24 hours of admission, C-reactive protein, and urea levels.

These predictors are biologically plausible and have been associated with worse clinical outcomes and mortality in acute pancreatitis in previous studies; for instance, age and multi‑organ dysfunction have been linked to increased mortality risk [18,19], while CRP has shown good prognostic accuracy in predicting mortality when measured at 24 hours [20], and urea levels have been consistently associated with poor outcomes [18]. Although both creatinine and urea are markers of renal function, urea remained independently associated with in-hospital mortality after multivariable adjustment, which may reflect its ability to capture not only renal impairment but also systemic dehydration, catabolic stress, and neurohormonal activation commonly observed in severe disease.

Early multi-organ dysfunction emerged as the strongest predictor of mortality, underscoring the critical role of early systemic involvement in adverse outcomes [21,22]. Elevated inflammatory markers and renal dysfunction, reflected by CRP and urea levels, likely capture both disease severity and physiological stress, while increasing age has consistently been associated with poorer outcomes in acute pancreatitis [23,24].

The derived risk score achieved good discrimination, with an area under the receiver operating characteristic curve of approximately 0.81 in the primary analysis. Internal validation using bootstrap resampling revealed minimal optimism, indicating limited overfitting. Temporal validation restricted to patients admitted during the later study period further supported the stability of model performance in a contemporary cohort. When applied to the study population, the risk score stratified patients into low-to-intermediate-risk and high-risk categories, with substantially higher observed in-hospital mortality in the high-risk group.

Recent studies have proposed alternative mortality prediction models in acute pancreatitis, often focusing on selected high-risk populations. For example, Chen et al. developed and internally validated a multivariable model for 30-day mortality among patients with severe acute pancreatitis admitted to the intensive care unit, reporting high discriminatory performance [25]. In contrast to that ICU-focused approach, the present study was conducted in a broader, unselected hospital cohort and targeted in-hospital mortality across the full spectrum of disease severity. While Chen et al. identified biochemical markers such as AST, ALP, triglycerides, and creatinine as key predictors, our model emphasizes early multi-organ dysfunction and routinely available inflammatory and renal markers obtained within the first 24 hours of hospitalization, allowing practical early inpatient risk stratification during this period.

Several prognostic tools for acute pancreatitis, including Ranson’s criteria, APACHE II, BISAP, and the revised Atlanta classification, are widely used in clinical practice [10,26]. However, many of these systems either require repeated assessments, extensive physiological and laboratory measurements, imaging-based variables, or reassessment at 24–48 hours, which may limit their applicability for early inpatient severity stratification within the first 24 hours of hospitalization [27].

Recent evidence further supports the growing role of clinically interpretable prediction tools in pancreatic disease. Song et al. developed a multicentre model for early prediction of infected pancreatic necrosis in patients with acute pancreatitis using routinely available variables collected within the first 24 hours of admission, including temperature, respiratory rate, calcium, blood urea nitrogen, and glucose [28]. Although their outcome differed from the present study’s endpoint of in-hospital mortality, both studies emphasize the feasibility of early inpatient risk stratification using readily available clinical information. More broadly, Sherpa et al. highlighted that prediction tools in pancreatic disorders require clear clinical applicability, transparent reporting, and external validation before routine implementation [29]. These considerations support the intended role of the present score as a practical early inpatient stratification tool that requires validation in independent cohorts before clinical use.

The present score was intentionally designed to rely exclusively on variables routinely available within the first 24 hours of hospitalization. Direct head-to-head comparison with established prognostic systems such as BISAP, Ranson’s criteria, or APACHE II was not performed, as these tools require variables collected beyond the early admission phase or repeated assessments that were not uniformly or prospectively available in the present dataset. Retrospective reconstruction of these scores would risk incomplete data capture and biased performance estimates. Rather than performing potentially misleading retrospective comparisons, the present model prioritizes feasibility, parsimony, and early applicability within real-world admission workflows. Accordingly, the primary advantage of the proposed score lies not in superior predictive accuracy, but in its simplicity and suitability for early inpatient risk stratification during the first 24 hours of hospitalization.

Several methodological strengths merit emphasis. This study includes a relatively large cohort of consecutive patients spanning multiple years, enhancing the representativeness of the study population. Missing data were handled using multiple imputation, reducing potential bias and preserving statistical power. In addition, model performance was evaluated using complementary validation approaches, including calibration assessment, bootstrap internal validation, and temporal validation, in accordance with TRIPOD recommendations for prediction model development.

Nevertheless, several limitations should be considered. Importantly, the proposed model is designed for early inpatient risk stratification using variables available within the first 24 hours of hospitalization and should not be interpreted as a point-of-triage prediction tool at emergency department presentation. Early multi-organ dysfunction within the first 24 hours was deliberately included as a predictor to enhance clinical utility during the initial hospitalization period rather than to serve as a strict baseline prognostic marker. This reflects an intentional balance between early physiological assessment and practical risk stratification, acknowledging that MODS may partially capture evolving disease severity. However, sensitivity analysis excluding early MODS demonstrated preserved discriminative performance (AUC 0.79), supporting that the model’s predictive ability is not solely driven by inclusion of this variable.

This was a single-center study, which may limit generalizability to other healthcare settings with different patient populations, pancreatitis etiologies, or management practices. Although internal and temporal validation demonstrated stable model performance, the absence of external validation in independent cohorts represents a key limitation. The original predictor-selection strategy may be susceptible to overfitting given the limited number of mortality events. Specifically, the 53 observed deaths among 11 evaluated candidate predictor parameters corresponded to 4.82 events per candidate predictor parameter, and the precision and stability of individual coefficient estimates should therefore be interpreted cautiously.

However, sensitivity analysis using ridge-penalized regression incorporating all candidate predictors demonstrated only modestly greater optimism-corrected discrimination and did not improve optimism-corrected overall predictive accuracy compared with the existing four-predictor model underlying the simplified score. In addition, categorization of CRP and urea in the simplified points-based score may result in some loss of predictive information compared with the underlying continuous regression model, reflecting a deliberate trade-off in favor of bedside usability. Calibration at higher predicted risk levels should also be interpreted carefully due to the limited number of high-risk patients and outcome events. Consequently, the proposed score should be considered a preliminary, clinically interpretable risk-stratification tool requiring external validation in independent, preferably multicenter cohorts before clinical implementation.

From an implementation perspective, the proposed score is intended to support structured risk reassessment during the first 24 hours of hospitalization, when its component variables are routinely available. Patients classified as higher risk may warrant closer clinical monitoring, earlier senior review, and increased vigilance for deterioration or need for escalation of care. However, the score should not be used as a stand-alone decision rule or guide management changes before external validation and prospective evaluation of its clinical impact.

Finally, the retrospective design precludes assessment of whether implementation of the score improves clinical decision-making or patient outcomes, which will require prospective evaluation. Despite these limitations, the model demonstrated consistent performance across complementary internal validation strategies, supporting its potential utility for early inpatient risk stratification.

## Conclusions

In conclusion, this study developed and internally validated a simple, admission-phase clinical risk score for predicting in-hospital mortality in patients with acute pancreatitis. The model demonstrated good discrimination, calibration, and clinical interpretability in internal analyses. External validation in independent, preferably multicenter cohorts is required before broader clinical implementation.

## Supporting information

S1 Checklist TRIPOD checklist. Completed TRIPOD checklist for the development and internal validation of the acute pancreatitis in-hospital mortality prediction model.
(DOCX)

S2 Early Inpatient Risk Score Tool Standalone scoring table, calculation instructions, and risk stratification criteria for the admission-phase in-hospital mortality prediction model. (DOCX)

S3 Data Anonymized dataset underlying the findings of this study. (XLSX)
