# Supplementary material for: Development and internal validation of an early inpatient risk score for in-hospital mortality in acute pancreatitis: A retrospective cohort study
Source: PLoS One. 2026 Jul 6;21(7):e0352980. doi: 10.1371/journal.pone.0352980 (PMC13336172; doi:10.1371/journal.pone.0352980)
Supplement: S1 Checklist — Completed TRIPOD checklist for the development and internal validation of the acute pancreatitis in-hospital mortality prediction model. (DOCX) [file pone.0352980.s001.docx]

| **TRIPOD Item** | **Description** | **Page / Section** |
| --- | --- | --- |
| **Title and Abstract** |  |  |
| 1 | Identify study as development and/or validation of a prediction model | Title |
| 2 | Structured abstract with objectives, methods, results, conclusions | Abstract |
| **Introduction** |  |  |
| 3a | Background and rationale for developing prediction model | Introduction |
| 3b | Objectives, including development and internal validation | Introduction |
| **Methods** |  |  |
| 4a | Study design and source of data | Study Design and Setting |
| 4b | Key study dates | Study Population |
| 5a | Study setting and number of centers | Study Design and Setting |
| 5b | Participant eligibility criteria | Study Population and Eligibility Criteria |
| 5c | Details of treatments (if relevant) | Not applicable |
| 6a | Definition of outcome | Outcomes |
| 6b | Blinding of outcome assessment | Not applicable (retrospective EMR data) |
| 7a | Definition of predictors | Predictor Variables |
| 7b | Blinding of predictor assessment | Not applicable |
| 8 | Sample size considerations | Sample Size Considerations |
| 9 | Missing data handling | Data Collection and Management |
| 10a | Statistical analysis methods | Statistical Analysis |
| 10b | Model-building procedures | Phase 2: Model Development |
| 10c | Model performance measures | Model Validation and Performance Assessment |
| 10d | Validation method | Internal Validation |
| **Results** |  |  |
| 11 | Participant flow and exclusions | Results; Fig 1 |
| 12 | Participant characteristics | Table 1 |
| 13a | Model development results | Tables 2 and 3 |
| 13b | Full model specification | Not reported for the underlying continuous prediction model; S2 File provides complete specification of the simplified points-based early inpatient risk score. |
| 14a | Model performance | Figures 2–4 |
| 14b | Validation results | Internal and Temporal Validation |
| **Discussion** |  |  |
| 15a | Model performance and interpretation | Discussion |
| 15b | Comparison with other models | Discussion |
| 16 | Study limitations | Discussion – Limitations |
| 17 | Clinical implications and future research | Discussion |
| Other Information |  |  |
| 18 | Funding | Funding statement |
| 19 | Ethics approval | Ethical Considerations |
