# Supplementary material for: Development and internal validation of an early inpatient risk score for in-hospital mortality in acute pancreatitis: A retrospective cohort study
Source: PLoS One. 2026 Jul 6;21(7):e0352980. doi: 10.1371/journal.pone.0352980 (PMC13336172; doi:10.1371/journal.pone.0352980)
Supplement: S2 Early Inpatient Risk Score Tool — (DOCX) [file pone.0352980.s002.docx]

**Early Inpatient Risk Score for In-Hospital Mortality in Acute Pancreatitis**

This clinical risk score was developed to support early inpatient risk stratification in adults hospitalized with acute pancreatitis using variables available within the first 24 hours of hospitalization.

This tool is distributed under the Creative Commons Attribution 4.0 International License (CC BY 4.0), which permits unrestricted use, distribution, and reproduction in any medium, provided the original work is properly cited.

**Scoring System**

Assign points according to the criteria below:

| Predictor | Criteria | Points |
| --- | --- | --- |
| Age | 1 point per 10-year increase (age in years divided by 10 and rounded down to the nearest whole number) | 1 point per decade |
| Early multi-organ dysfunction (MODS <24 h) | Present (modified Marshall score ≥2 in ≥1 organ system during the first 24 hours of hospitalization) | 4 |
| C-reactive protein (CRP) | ≥100 mg/L | 2 |
| Urea | ≥8 mmol/L | 2 |

**Score Calculation**

Total score = Sum of all assigned points.

Observed score range in the development cohort: 1–15 points.
The theoretical maximum score is not fixed, as age contributes incrementally without an upper cap within the model structure.

**Risk Stratification**

Patients may be stratified into the following risk categories:

- Low-to-intermediate risk: ≤5 points.
- High risk: ≥6 points.

In the development cohort:

- Low-to-intermediate-risk group: 1.8% in-hospital mortality.
- High-risk group: 8.8% in-hospital mortality.

Observed mortality rates reflect the development cohort and may vary in external populations.

**Intended Use**

This score is intended for early inpatient risk stratification using variables routinely available within the first 24 hours of hospitalization.

It is not intended for emergency department triage prediction and should not replace clinical judgment.
